# Can Artificial Intelligence Enhance Syncope Management? A *JACC: Advances* Multidisciplinary Collaborative Statement

**DOI:** 10.1016/j.jacadv.2023.100323

**Published:** 2023-05-10

**Authors:** Giselle M. Statz, Aron Z. Evans, Samuel L. Johnston, Mehul Adhaduk, Avinash R. Mudireddy, Milan Sonka, Sangil Lee, E. John Barsotti, Fabrizio Ricci, Franca Dipaola, Madeleine Johansson, Robert S. Sheldon, Venkatesh Thiruganasambandamoorthy, Rose-Anne Kenny, Tyler C. Bullis, Deepak K. Pasupula, Jon Van Heukelom, Milena A. Gebska, Brian Olshansky

**Affiliations:** a Division of Cardiovascular Medicine, Roy J. and Lucille A. Carver College of Medicine, University of Iowa, Iowa City, Iowa, USA; b Department of Internal Medicine, Roy J. and Lucille A. Carver College of Medicine, University of Iowa, Iowa City, Iowa, USA; c The Iowa Initiative for Artificial Intelligence, University of Iowa, Iowa City, Iowa, USA; d Department of Emergency Medicine, Roy J. and Lucille A. Carver College of Medicine, University of Iowa, Iowa City, Iowa, USA; e Department of Epidemiology, College of Public Health, University of Iowa, Iowa City, Iowa, USA; f Department of Neurosciences, Imaging and Clinical Sciences, Institute for Advanced Biomedical Technologies, University G. d’Annunzio, Chieti, Italy; g Internal Medicine, Syncope Unit, IRCCS Humanitas Research Hospital, Humanitas University, Rozzano, Milan, Italy; h Department of Cardiology, Skåne University Hospital, Lund University, Malmo, Sweden; i Department of Cardiac Sciences, University of Calgary, Calgary, Alberta, Canada; j Department of Emergency Medicine, University of Ottawa, Ottawa, Ontario, Canada; k Department of Medical Gerontology, School of Medicine, Trinity College, Dublin, Ireland; l Division of Cardiovascular Disease, Department of Internal Medicine, MercyOne North Iowa Heart Center, Mason City, Iowa, USA

**Keywords:** artificial intelligence, emergency department, machine learning, syncope, transient loss of consciousness

## Abstract

Syncope, a form of transient loss of consciousness, remains a complex medical condition for which adverse cardiovascular outcomes, including death, are of major concern but rarely occur. Current risk stratification algorithms have not completely delineated which patients benefit from hospitalization and specific interventions. Patients are often admitted unnecessarily and at high cost. Artificial intelligence (AI) and machine learning may help define the transient loss of consciousness event, diagnose the cause, assess short- and long-term risks, predict recurrence, and determine need for hospitalization and therapeutic intervention; however, several challenges remain, including medicolegal and ethical concerns. This collaborative statement, from a multidisciplinary group of clinicians, investigators, and scientists, focuses on the potential role of AI in syncope management with a goal to inspire creation of AI-derived clinical decision support tools that may improve patient outcomes, streamline diagnostics, and reduce health-care costs.

## Highlights

• Syncope remains a complex, difficult to manage condition associated with adverse cardiovascular outcomes.
• Artificial intelligence may assist in diagnosis, risk stratification, and management decisions, yet challenges remain.
• Prospective, multicenter, and multidisciplinary datasets could serve as the ideal machine learning platform.
• Further studies should compare machine learning approaches to existing risk stratification tools and clinical judgment.

## Syncope: the challenge

**Figure 1:**
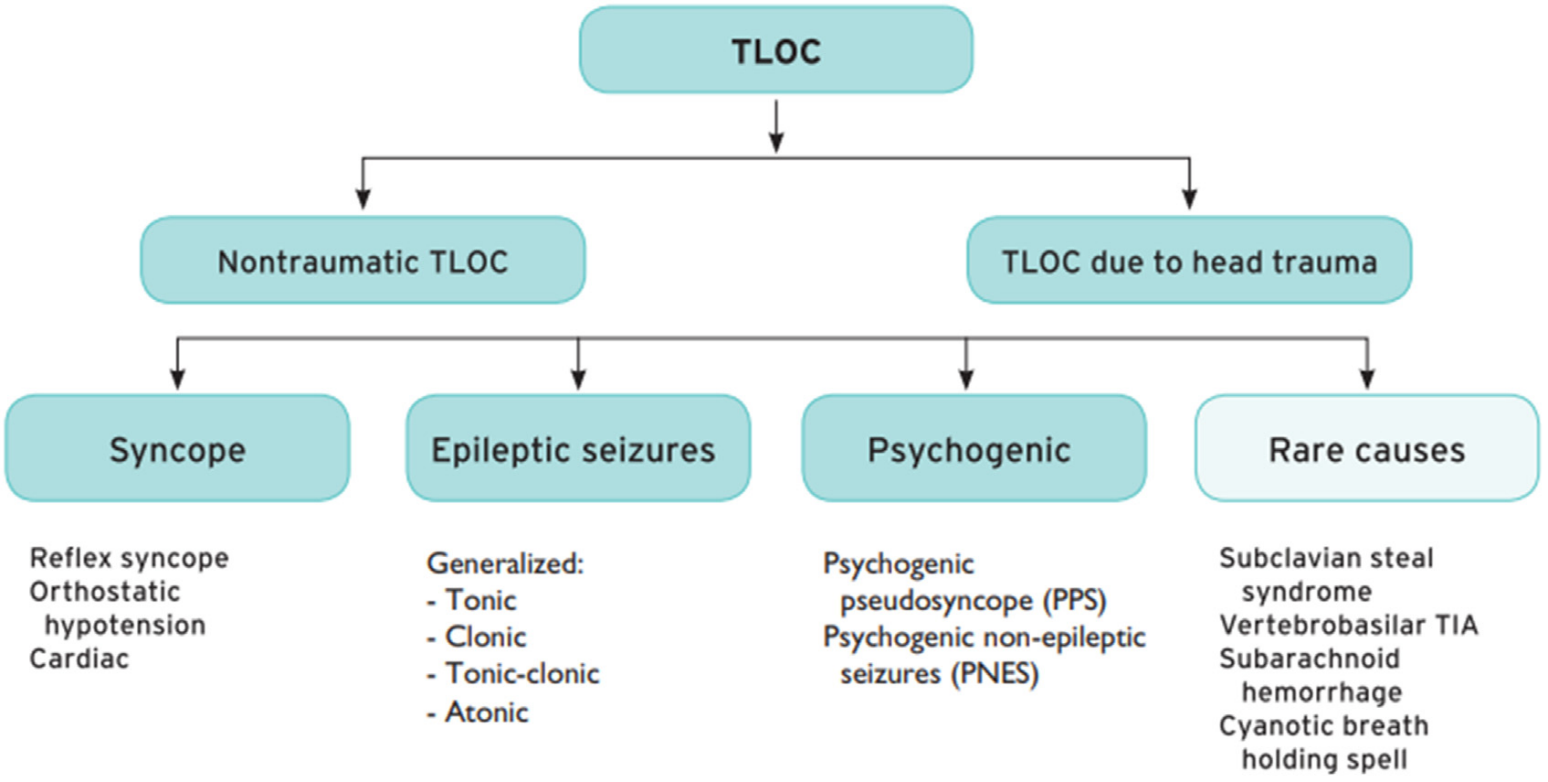
**Syncope in the Context of Transient Loss of Consciousness** Adapted from the 2018 European Society of Cardiology Guidelines for the Diagnosis and Management of Syncope.^3^ TIA = transient ischemic attack; TLOC = transient loss of consciousness.

Syncope, a form of transient loss of consciousness (TLOC) followed by rapid, complete recovery,^1^ remains a consequential medical problem.^2^ Current guidelines^1^^,^^3^ stress the importance of distinguishing syncope from other forms of TLOC and altered states of consciousness including head trauma, seizures, drug overdoses, and psychogenic or metabolic causes (Figure 1). Once syncope is suspected, identifying the mechanism and cause are crucial for further management. However, reliance on history, physical examination, and clinical acumen may not be enough.^1^^,^^4^ The history from patient recollection and observations from witnesses, if present, may be difficult to interpret.^5^

A spectrum of causal and associated conditions, ranging from benign vasovagal faints to life-threatening arrhythmias and other cardiopulmonary conditions, further complicate assessment. Up to 10% of patients presenting to the emergency department (ED) with syncope will have serious outcomes over the short-term; identifying that high-risk population remains a primary focus.^3^ Age is a nonspecific discriminator. Older individuals may simply be at risk from concomitant, unrelated, and life-threatening conditions even if syncope itself is benign. Occasional younger individuals are at a continued risk of life-threatening collapse.^6^ ED physicians, therefore, face substantial dilemmas in risk stratification and triage,^6^^,^^7^ particularly for those at “intermediate risk.”^1^^,^^3^

**Figure 2:**
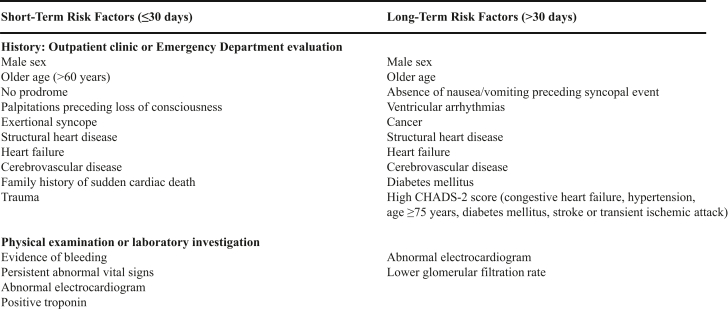
**Short- and Long-Term Risk Factors in Syncope Patients** Adapted from the 2017 ACC/AHA/HRS Guideline for the Evaluation and Management of Patients with Syncope.^1^

Syncope risk scores were developed to help provide a uniform methodology to help risk stratify patients with syncope. The syncope risk calculators that have been developed (Supplemental Appendix) are not clearly better than good clinical assessment.^8^ These tools have not definitively improved guidance in predicting short- and intermediate-term risk (Figure 2) of serious outcomes. Further, these risk scores are not designed to determine diagnosis, risk of recurrence, or benefit of hospitalization. Indeed, these decision rules predict outcomes based on comorbidities rather than syncope itself. American and European guidelines give Class IIb recommendations for these tools.^1^^,^^3^

Designated ‘syncope units’ expedite diagnoses, reduce admissions, and improve outcomes.^9^ However, regional and international differences in resources, training of personnel, and health-care delivery systems remain barriers.^9^^,^^10^ Their utility is dependent upon a clinician who is expert in syncope management.

Current diagnostic and risk assessment strategies remain variable and imprecise. Despite technological advances, including novel ambulatory electrocardiography (ECG) monitoring strategies, establishing a definitive diagnosis in patients with syncope remains largely dependent on collecting a careful patient history (highly variable among physicians), but this is low yield, and many patients remain undiagnosed. Over many decades management has not changed significantly.

Artificial intelligence (AI) and machine learning (ML) may help address some of these issues (Table 1). Preliminary AI-based risk stratification and diagnostic methods are encouraging,^11^ and include predicting short-term adverse events^12^^,^^13^ and hospitalization,^14^^,^^15^ diagnosing vasovagal syncope,^16^^,^^17^ differentiating syncope from other forms of TLOC,^18^ assisting in ECG interpretation,^19^ interpreting ambulatory ECG monitors and implantable loop recorders,^19^ and reviewing records via natural language processing (NLP).^20^ However, the ultimate role for AI in syncope management remains undeveloped. Potential AI solutions could assist with integrating multiple data inputs, discover unusual associations between risks and diseases, improve diagnostic capabilities, forecast prognoses, predict treatments, and standardize syncope care.

**Table 1:** How AI Could Improve Syncope Management

Purpose	Clinical Question
Define the event	Is it syncope or another cause of TLOC?
Diagnose the underlying etiology	What is the cause of syncope?
Risk stratification	Is the patient at risk of short- and long-term adverse outcomes?
Predict recurrent events	How likely is the patient to have syncope again?
Extract clinical info from ECG findings	Does the ECG indicate a cause for syncope?
Determine appropriate ED disposition	Hospitalization or discharge?
Assess the need for immediate interventions	Acute treatment or chronic assessment?
Evaluate the need for diagnostic tools	Is TTE, cardiac catheterization, or telemetry needed?
Determine long-term management strategies	What is the optimal treatment and follow-up?

The key objectives and clinical questions that potentially could be addressed with AI. Examples of short- and long-term adverse outcomes are described in Table 4.

AI = artificial intelligence; ECG = electrocardiogram; ED = emergency department; TLOC = transient loss of consciousness; TTE = transthoracic echocardiography.

The goal of this review is to focus on the potential application of AI to improve syncope management, including fundamental AI and ML concepts, potential clinical endpoints, usable datasets, challenges, and solutions. Major themes for future AI syncope projects are listed in Table 2.

**Table 2:** Major Themes in Developing Future AI-Based Syncope Projects

AI could assist clinicians in separating true syncope from other forms of TLOC.
For true syncope cases, AI could assist in diagnosing the underlying etiology and differentiating benign from life-threatening causes.
AI may help identify patient characteristics and comorbidities that affect short- and long-term outcomes (eg, 30-d mortality, recurrent episodes, sudden death, total mortality, AND rehospitalization).
Accurate features (ie, input variables) and labels (ie, output variables) are necessary for supervised ML.
A global, multicenter, and multidisciplinary approach is needed, and a prospective dataset is ideal. Existing retrospective health-care datasets are inconsistent and imperfect from a ML perspective.
AI is a promising, wide-reaching clinical tool, but expectations for its ability to facilitate assessment, triage, and management of syncope patients must be delineated.

This table summarizes the key themes in this review article.

AI = artificial intelligence; ML = machine learning; TLOC = transient loss of consciousness.

## Understanding AI and ML

AI is revolutionizing health care.^21^, ^22^, ^23^ AI in some ways is capable of mimicking human cognitive function. ML, a subset of AI, uses input data to train decision-making models that improve through experience^24^^,^^25^ and excel when applied to large data sets.^26^ An introduction to AI begins with a glossary of ML terms (Table 3).

**Table 3:** Glossary of Machine Learning Terms

Artificial Intelligence (AI)	Intelligence demonstrated by a nonhuman program capable of solving complex tasks.
Artificial neural network (ANN)	A type of DL model which contains an input, output, and any number of hidden layers.
Black box models^a^	ML models that are complex, highly non-linear, and whose inner workings are not easily interpretable (e.g., DL models). Relationships between inputs and outputs cannot be explained. These models are usually more accurate than white box models, but their lack of explainability and risk of overfitting or spurious correlations are disadvantageous.
Classification model	Supervised ML method which uses features (ie, input data) to predict (or classify) labels. Subtypes include binary and multi-class classification.
Class imbalance	When a dataset has a disproportionate number of labels within a classification problem.
Cluster analysis	Unsupervised ML method that maps similar data samples into groups. Their significance is then defined by a human observer.
Deep learning (DL)	A subset of ML that uses a neural network containing multiple interconnected layers intended to mimic the wiring of the human brain.^26^
Ensemble learning	When multiple independent ML models are combined to create averaged predictions, which frequently perform better than single ML methods in isolation.
Features	Input variables to a ML model.
Feature importance ranking	A ML tool used to identify the relative influence each feature has on the chosen outcome.
Labels	Output variables from a ML model.
Machine learning (ML)	A subset of AI defined as a program that uses a predefined process to learn structures and patterns in data without human involvement.
Natural language processing (NLP)	ML method of interpreting typed or spoken language and extrapolating its meaning.
Overfitting	When a ML model becomes overtrained on one set of data and is not generalizable to another set.
Regression model	ML method that predicts a continuous outcome value. Common subtypes include linear and logistic regression, though DL can be used.
Supervised machine learning	Training a model using labeled data, analogous to learning from a teacher who provides the questions and correct answers.
Training, validation, and testing datasets	Partitioned subsets of a dataset that are separately used to train the model, validate its predictive ability, and then test its generalizability to unseen data.
Unsupervised machine learning	Training a model to uncover patterns and structures in an unlabeled dataset. A common example is cluster analysis.
Upsampling	A ML training tool that addresses class imbalance by expanding the more infrequent class to provide the ML model a more even number of examples to learn from. “Downsampling” of the more frequent class may also be used in a similar fashion.
White box models^a^	ML models that easily demonstrate how they produce predictions and which input features are influential (eg, linear regression, decision trees). However, they are often less accurate than black box models because they assume linear relationships between inputs and outputs, which is rarely true in reality.

Definitions of common ML concepts, adapted from those listed in the Google Developers Machine Learning Education Glossary.^24^ Portions of this page are modifications based on work created and shared by Google and used according to terms described in the Creative Commons 4.0 Attribution License.

AI = artificial intelligence; ANN = artificial neural network; DL = deep learning; ML = machine learning; NLP = natural language processing.

a These terms were defined by A.R.M., AI expert author.

**Figure 3:**
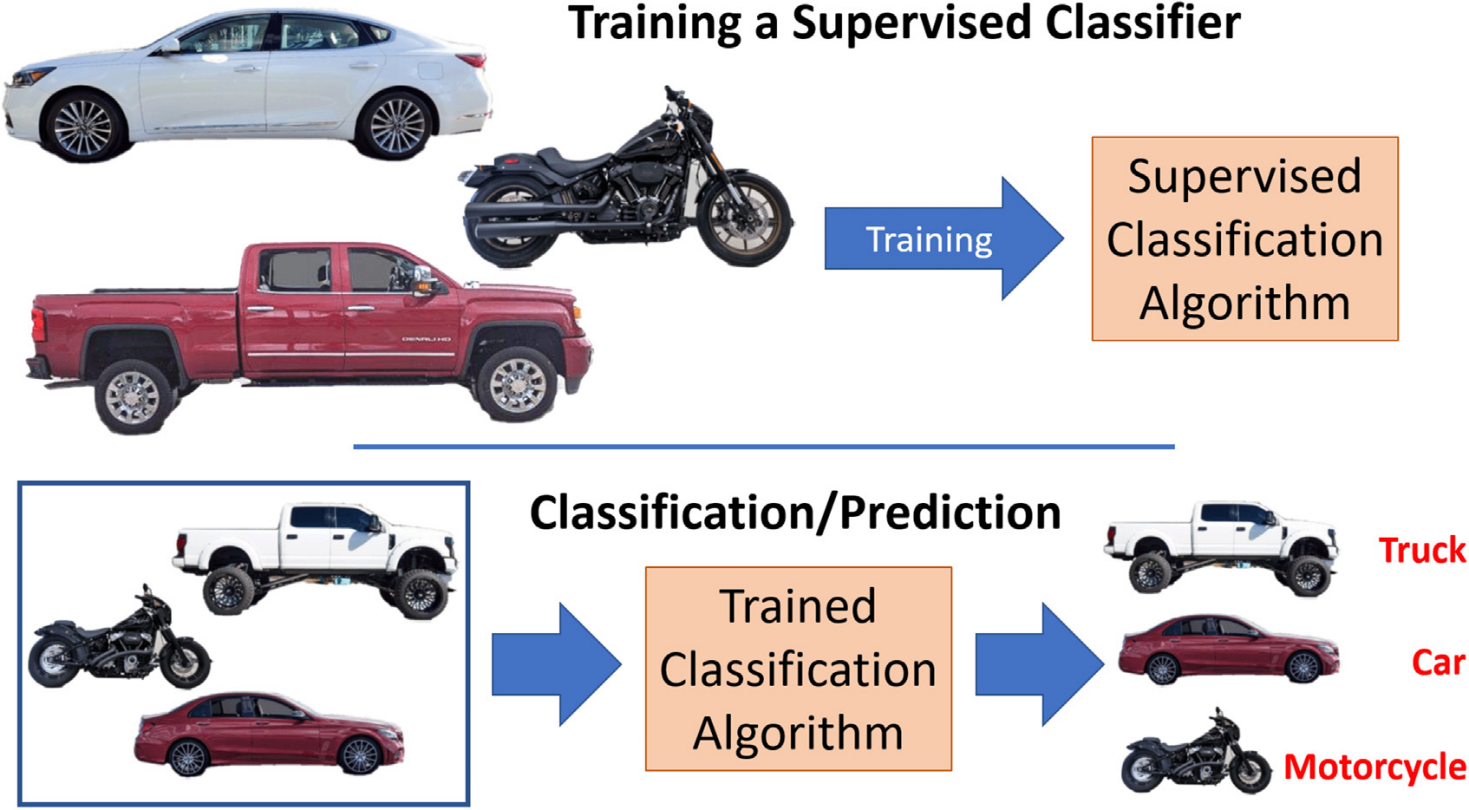
**Steps Involved in Supervised Machine Learning** **(Top row)** A supervised classifier is trained on different types of vehicles (eg, car, truck, and motorcycle). **(Bottom row)** When given a new set of vehicles of different colors/model types, the trained classifier can assign the correct vehicle label.

The type of ML most relevant to health care presently is “supervised learning” where correct answers are provided by a “teacher” supervising the learning process, analogous to teaching a child to recognize common street vehicles (Figure 3). This requires a training set of objects which consists of “features” or descriptors, as well as “classification labels” or answers. For example, 2 wheels = motorcycle, 4 wheels = car. The learning process is iterative with the objective being to teach the ML algorithm or “classifier” to maximize the number of correct decisions while minimizing incorrect ones. Once the classifier is effectively trained on a “training dataset,” its performance and generalizability is assessed on a “testing dataset” of never-before-seen data samples. A common problem in supervised ML is “overfitting,”^27^ when the algorithm is too specific for the training dataset and cannot be applied to a testing dataset. For example, if the training dataset is too small and all cars happened to be red, it may not accurately label cars of different colors.

If classification labels are not defined to guide the learning process, then “unsupervised learning” can be considered. In unsupervised learning, ML groups data samples into clusters based on a predefined similarity score. Several models exist for such an approach, with algorithm choice dependent on the use.^28^^,^^29^ Traditional applications of unsupervised learning models are dimensionality reduction, association rule mining, and cluster analysis.^28^^,^^29^ Cluster analysis has been used across medical disciplines for several decades.^30^, ^31^, ^32^, ^33^ Clusters, however, do not have classification labels since that information is not available during the learning process. Thus, clusters can be created, but understanding the meaning can be a complex process pertaining to 2 main questions: 1) how many clusters should be used; and 2) is there real-world relevance behind the formed clusters? Mathematical formulations such as the elbow method can determine the best number of clusters,^34^ but for real-world relevance and cluster significance, additional descriptive, quantitative, and more advanced statistical approaches may be needed.^29^^,^^35^^,^^36^ Popular algorithms, such as K-means clustering and hierarchical clustering can be used to perform neuroimage segmentation,^33^ find similarities among breast cancer patients,^37^ and phenotype pediatric patients with inflammatory bowel disease.^35^ For syncope patients, unsupervised learning may yield insights such as identifying new patient clusters with novel risk factors or similar responses to treatments, particularly because the correct label may not be known definitively.

Despite the practicality and effectiveness of traditional ML algorithms, “deep learning” receives widespread attention. Deep learning neural networks are characterized by a structure of interconnected layers inspired by the wiring of the human brain.^26^ Their ability to learn often exceeds that of other ML approaches, but they are not free of limitations, including reliance on large training sets, sensitivity to training set composition, and “black box” characteristics that escape human understanding. The inability for one to understand how the computer arrives at a solution (ie, the black box) forces the clinician to accept results on blind faith. This can lead to overconfidence and overreliance on the computer algorithm, and erroneous conclusions with clinically significant ramifications. Ultimately, the clinician must decide whether the computer output is reasonable and safe. Just as the clinician may order a laboratory test that yields an incorrect result, the output from an ML algorithm may also be in error. In both cases, the clinician must make the final call and cannot trust the results blindly. This is also why AI-based clinical research must be externally validated with randomized control trials prior to widespread application.

## Clinical endpoints for ML

Training supervised ML programs require large datasets containing accurate features (ie, inputs) and labels (ie, outputs), so that correct learning can occur. In health care, ML programs may receive patient-specific features (eg, symptoms, vital signs, and test results) and associate these with certain labels (eg, diagnoses, treatments, and adverse events). These features and their associated labels can be compiled into database format, which can then be divided and used in training, validation, and testing stages. For syncope, it is paramount to identify which clinical questions ML can answer. These could be diagnostic (what caused syncope?), prognostic (what subsequent adverse event occurred?), or management related (did triage, testing, or treatment affect outcome?). Table 1 lists proposed clinical endpoints (eg, event definition, diagnosis, risk assessment, and ED disposition) that, if better predicted by a ML model, could improve patient care.

A ML model that predicts which TLOC patients had syncope could be helpful. The ambiguous nature of their presentations (especially in elderly patients) and uncertainty of their diagnoses make it difficult to gather the correct labels required for ML. The absence of any universal gold standard test means the diagnosis may never be known with certainty. Even when the cause is known, confusion may exist regarding the mechanism. Transient asystole can be a manifestation of sinus node disease or simply be a vagal response. Further, a vagal response can be a singular event (eg, sight of blood) or a repetitive problem (eg, temporal lobe seizures or idiopathic). These diagnostic uncertainties illustrate the complexities of TLOC and syncope, which can lead to incorrect labels and affect the clinical application of ML.

The importance of risk stratification in syncope patients cannot be overstated. The American College of Cardiology (ACC)/American Heart Association (AHA) and European Society of Cardiology (ESC) guideline committees compiled short- (<30 days) and long-term (>30 days) adverse events associated with syncope^1^^,^^3^ (Figure 2). As opposed to diagnostic outcomes, these adverse events are definitive, reportable, and already recognized in risk stratification studies (Supplemental Appendix), and therefore, more suitable endpoints for supervised ML algorithms. Table 4 summarizes endpoints derived from previous risk stratification studies. However, it is critical to ensure that outcomes are syncope specific. For example, short-term adverse outcomes^38^ could overlap with nonsyncopal causes of TLOC (eg, stroke).

**Table 4:** Endpoints of Interest From Syncope Risk-Stratification Studies

	Short-Term Risk Endpoints (<30 d)	Long-Term Risk Endpoints (>30 d Up to 1 y)
Clinical endpoints specific to syncope	• Death • Myocardial infarction • Life-threatening arrhythmia • Pulmonary embolism • Return to ED • Early readmission • Falls • Trauma • Hemorrhage requiring transfusion of at least 2 U of packed red blood cells	• Death • Cardiopulmonary resuscitation • ICU admission
Procedural endpoints specific to syncope	• Pacemaker or defibrillator placement • Percutaneous coronary intervention • Cardiopulmonary resuscitation • Carotid artery interventions	• Pacemaker or defibrillator placement
Clinical and procedural endpoints not specific to syncope	• Cerebrovascular accident • Subarachnoid hemorrhage • Acute surgery or endoscopic intervention	• Antiarrhythmic therapy

This table summarizes the potential endpoints derived from previous risk stratification studies (Supplemental Appendix) that could serve as predictable outputs in a machine-learning algorithm.

ED = emergency department; ICU = intensive care unit.

To simplify issues of clinical complexity, AI could focus on one element in isolation, such as the ECG. The ECG provides fairly objective information that can diagnose life-threatening cardiogenic causes of syncope. As mentioned earlier, AI-enhanced ECG interpretation is already in early development.^19^^,^^39^ Focus on this objective measure could allow improved oversight after an unreliable subjective history. The widespread use of wearable devices can also provide data for otherwise subjective events.^40^^,^^41^ ML algorithms have been applied to wearable devices to detect generalized tonic-clonic seizures with high sensitivity.^42^ ML has the advantage of analyzing large amounts of data, a task that would otherwise be overwhelming and time consuming.

Predicting future adverse events can help with immediate management decisions, including discharge strategy. After extracting key historical and risk-defining features, physicians may err on the side of admission in abundance of caution. While it is crucial to avoid discharging a high-risk patient without proper treatment, health risks and costs from inappropriate admissions should also be considered. Choosing the best disposition from the ED is a multifaceted decision point that deserves attention in future ML-based syncope studies.

Syncope recurrence is another important endpoint. ML may predict who is at greatest risk for recurrence or readmission. In addition to improving treatment strategies and cost-conscious care, it could improve patient counseling. While providers focus on adverse outcomes, patients may be more concerned with recurrence, even if the cause is benign. Compared to other outcomes, which are difficult to extract from electronic medical record (EMR) datasets, recurrent syncope may be easier to account for because data on patients returning to the ED with a diagnosis of syncope are often available (assuming they present to the same facility or network). This could also better-predict hospital length of stay, a key metric for health-care costs and resource allocation.

The effectiveness and applicability of the ML algorithm depends on the quality of data provided as well as the algorithm chosen. A common challenge with clinical endpoints in syncope is a lack of reliable classification labels (eg, inaccurate diagnosis codes, unavailable follow-up data). However, precise knowledge of these labels, while optimal, may not be totally necessary. Unsupervised learning can be considered if defined outcomes are not available; this type of learning is only useful for identifying imaginative patterns through clusters or broad associations. Alternatively, multiple ML decision makers can be employed (eg, cascading weak classifiers, boosting, or ensemble approaches), which can enhance overall performance, but this does not address imperfect labels directly. Similarly, through multicenter human collaboration, we can compile many real-life patient examples and their associated features and labels, and combine them into a consensus dataset for uniform agreement.

## Usable datasets

Reliable datasets containing accurate features and labels are ideal for ML, but current EMR databases can be inconsistent, incomplete, or inaccurate.^43^ No matter how good the clinical history and assessment, if documentation is faulty, classification labels may be inadequate. For example, labels may include diagnosis codes that provide incomplete information. Narrative information from the provider note is often more specific and accurate, thus AI-based NLP tools can be used to automatically extract and label elements from massive volumes of raw textual data. Moreover, to predict adverse outcomes using ML, it is important for the training set to have access to follow-up data after discharge. Some patients die or suffer adverse events that are not reported back to the ED. Similarly, some patients may need to see syncope specialists in the clinic and have follow-up tests (eg, tilt table testing) to achieve a correct diagnosis and treatment plan. Capturing this information requires data from ED, inpatient, and outpatient encounters, and is essential for correct decision labeling.

Retrospective national datasets, which have proven useful in epidemiologic studies, pose challenges for ML. The authors attempted to evaluate patients through the United States National Emergency Department Sample, which includes approximately 25 million syncope patients presenting to the ED over 10 years. This database captures patient-specific events via International Classification of Diseases codes. Information regarding ED visits and subsequent inpatient stays is provided; however, there is no information about events transpiring after discharge, making it difficult to predict any meaningful clinical outcomes. Likewise, other large administrative databases in the United States, though useful in many areas of medical research, are insufficient for ML-based analysis of syncope patients. Publicly funded datasets in other countries are beset by similar problems. Denmark, for example, has access to 5 million patients’ data, including diagnoses and prescriptions, but ECGs, blood pressures, and laboratory testing are not available, providing insufficient granularity for ML. Several other datasets show potential for data mining, such as the Medical Information Mart for Intensive Care-IV, which contains deidentified data from ED and intensive care unit encounters.^44^ Other institutional data may also be available upon request through internal mechanisms.^45^

Considering the inadequacies of current retrospective datasets, the creation of prospective, multicenter, and multinational datasets involving EDs, hospitals, ambulatory clinics, and syncope units is desirable. The clinical objectives and proposed steps of such a collaborative AI project are outlined in the Central Illustration. The dataset would utilize a prespecified, expert-adjudicated data collection process from which precise features and labels could be determined and verified. It would be committee-based for agreement across continents. This could be an ideal dataset on which to train a robust supervised ML algorithm. While it will be an enormous undertaking, its creation will be necessary. Early attempts are exemplified in the work of Grant et al,^13^ who prospectively collected multicenter cohort data from 8,000 patients and found that ML models matched the Canadian Syncope Risk Score in predictive performance of 30-day adverse outcomes. Additional datasets of such size and granularity will be needed to compare ML approaches with current clinical practice.

**Central Illustration:**
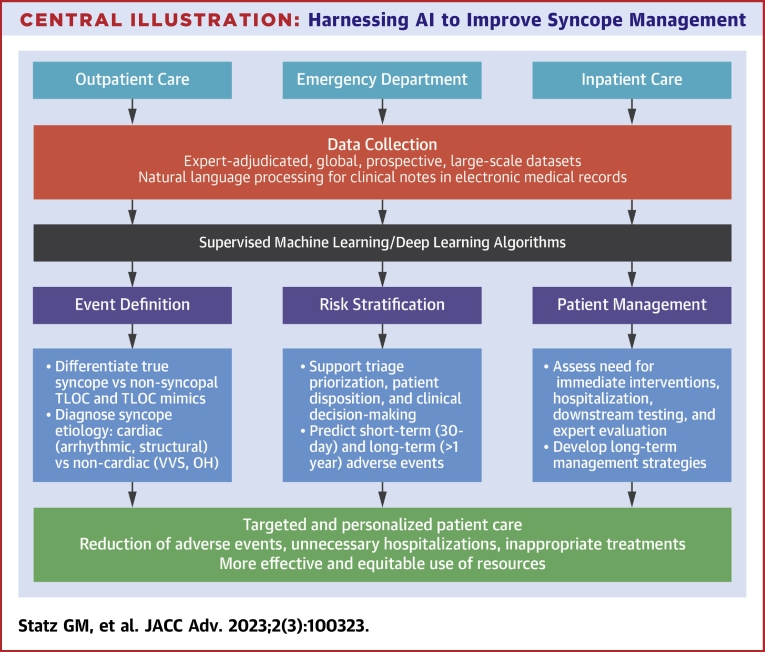
**Harnessing AI to Improve Syncope Management** This diagram outlines clinical objectives and proposed steps of AI-based initiatives to improve syncope management. Created with Biorender.com. AI = artificial intelligence; OH = orthostatic hypotension; TLOC = transient loss of consciousness; VVS = vasovagal syncope.

## Choosing a ML approach

There is no universal ML method across all tasks. Choosing an algorithm depends on various factors such as the goal of the study (eg, supervised, unsupervised), nature of the dataset (eg, linear vs non-linear, continuous vs categorical values, degree of incompleteness, and presence of bias), and computational or resource constraints. Specific ML tasks must be clarified, such as binary decision problems (eg, should a syncope patient be admitted or not?), multi-class decision problems (eg, what is the cause of syncope?), or regression problems (eg, what is the patient’s calculated risk score value?). Once these functions are established, several ML methods are chosen, tested on a mutual dataset, and the best performing method is selected. Alternatively, multiple methods may be used jointly in an ensemble approach, which frequently outperforms individual ML methods.

For supervised learning, many ML methods are available. One popular tool for ML design, SciKit Learn,^46^^,^^47^ lists >100 traditional ML training methods from which to choose. Some popular tools for deep learning include Tensor Flow, Keras, and Pytorch.^48^, ^49^, ^50^ Detailed description of ML methods is out of scope for the current article.

## Challenges and solutions

There are several challenges to using AI in syncope management, but solutions exist (Table 5). Some issues are inherent to syncope; others are universal to health-care datasets. Tackling these areas requires sensible dataset construction and collaboration between clinicians and data scientists.

**Table 5:** Challenges and Potential Solutions in Using AI for Syncope

Challenges	Potential Solutions
Identifying syncope and its underlying cause relies on a subjective history	Use natural language processing tools to mine information from unstructured data sources (eg, clinical notes)
There is no gold standard for syncope	Use prospective datasets, focus only on basic diagnostic distinctions (eg, vasovagal syncope, orthostatic hypotension)
Electronic medical records often contain inaccurate information not suitable for supervised ML	Identify reliable features and known correct labels that are well-documented, use prospective datasets, and apply ML techniques (eg, ensemble approaches)
Adverse cardiovascular outcomes occur in a minority of patients, resulting in imbalanced classification	Utilize larger datasets; apply ML techniques (eg, upsampling, downsampling)
Low-, intermediate-, and high-risk is difficult to define, personalized outcomes must be clarified	Perform phenotypic profiling via unsupervised ML (eg, cluster analysis)
Predicting short- and long-term adverse events requires follow-up data	Utilize data from EDs, hospitals, ambulatory clinics, and syncope units
Syncope is a ubiquitous clinical entity that spans multiple settings and demographics	Develop multidisciplinary, multicenter, and international collaborations
Retrospective health-care datasets are imperfect	Use expert-validated prospective datasets
AI is complex; expectations may be inaccurate	Collaborate with AI experts
AI may cause medicolegal and ethical dilemmas relating to patient autonomy, safety, and privacy	Educate physicians and beware of AI-related clinical risks; collaborate with medical ethics experts

The main challenges and potential solutions in using AI to improve syncope management.

AI = artificial intelligence; ED = emergency department; ML = machine learning.

A critical challenge to apply supervised ML to clinical data is data incompleteness. In addition to insufficient feature and label collection, data may be clinically irrelevant (eg, administrative datasets) or constrained by the data available at a given timepoint (eg, clinicians modify their decision-making with arrival of new test results). The data collection process itself is also limited by the capabilities of the facility where it occurs. These challenges highlight the importance of evaluating potential datasets in terms of feature/label reliability and their suitability for ML. Prospective approaches can ensure datasets are tailor-fit for ML on the front-end, if large enough cohorts can be obtained.

Defining the event or diagnosing the cause of syncope continues to rely on a subjective history from the patient or witness. NLP is likely to help incorporate some of this meaningful information. Dipaola et al^20^ developed an NLP algorithm using chart review and was able to locate 571 syncope patients from over 30,000 separate EMRs.

Using ML to diagnose the variety of causes for TLOC and syncope could be made easier through more simplified approaches, such as focusing on only basic distinctions (eg, vasovagal syncope vs orthostatic hypotension). Hussain et al^16^ employed a support vector machine model capable of using patient vital signs during the head-up tilt test to diagnose vasovagal syncope. Raphan et al^17^ developed a ML approach to identify vasovagal responses in an animal model during tilt table testing. Wardrope et al^18^ used patient and witness questionnaires to develop a ML model that accurately predicted the diagnosis in 86% of 249 patients known to have syncope, epilepsy, or psychogenic nonepileptic seizures. If correct labels can be achieved, ML techniques such as feature importance ranking^51^ can assess the relative contribution of patient symptoms, vital signs, or lab results toward achieving the correct diagnosis. There are many ML approaches used for feature importance ranking, (eg, gain, coverage, and permutation importance) and now deep-learning approaches are emerging.^52^

Risk stratification can be assessed if follow-up data are obtained, which depends on gathering clinical data from both inpatient and outpatient encounters. Because short-term syncope-specific adverse events are rare, robust statistical inferences can only be made from large cohorts of individuals and a sufficient number of adverse events. This disproportionate outcome data, termed “class imbalance,” is problematic for supervised ML approaches. Specific ML methods such as “upsampling” can help deal with imbalanced data at the training level. When upsampled, the more infrequent or minority class (eg, syncope patients with adverse outcomes) can be expanded to provide the ML model a more even number of examples from both classes. Downsampling of the majority class can also be done. Despite these challenges, the latest syncope AI projects exploring risk stratification are encouraging.^11^ Costantino et al^12^ used artificial neural networks (ANNs) and prospective datasets to predict short-term (<7-10 days) adverse events after syncope and found them comparable, if not superior, to current risk stratification tools, though not via direct head-to-head comparison. Based on the same data used to develop the Canadian Syncope Risk Score, Grant et al^13^ developed 4 ML models to predict short-term (<30 day) adverse outcomes after ED disposition that matched the Canadian Syncope Risk Score in performance. Prospective studies are needed to compare ML approaches to existing risk stratification tools and clinical judgment.

Compounding the challenges with risk assessment, specifying the definition of low-, intermediate-, and high-risk patients in syncope remains controversial. While underlying cardiovascular comorbidities augment risk, their relationship to syncope and how syncope itself affects outcomes that are age-, gender-, and disease-dependent. How to integrate these observations effectively into a robust, but useful, model for all disease entities has evaded clinicians. It is possible that unsupervised cluster analysis with phenomapping techniques^53^^,^^54^ could help identify distinct subtypes or phenotypes within the syncope population, and unique risk or prognostic profiles could be explored to quantify a patient’s risk of adverse outcomes.

In addition to predicting adverse outcomes, understanding hospitalization predictions can be useful. Falavigna et al^14^ used an ANN model that predicted hospitalization with a sensitivity of 100% and specificity of 79%, which outperformed the Osservatorio Epidemiologico sulla Sincope nel Lazio score and San Francisco Syncope Rule score. Lee et al^15^ also utilized an ANN approach to predict short (≤48 hours) vs long (>48 hours) hospital length of stay with an area under the curve of 0.81.

**Figure 4:**
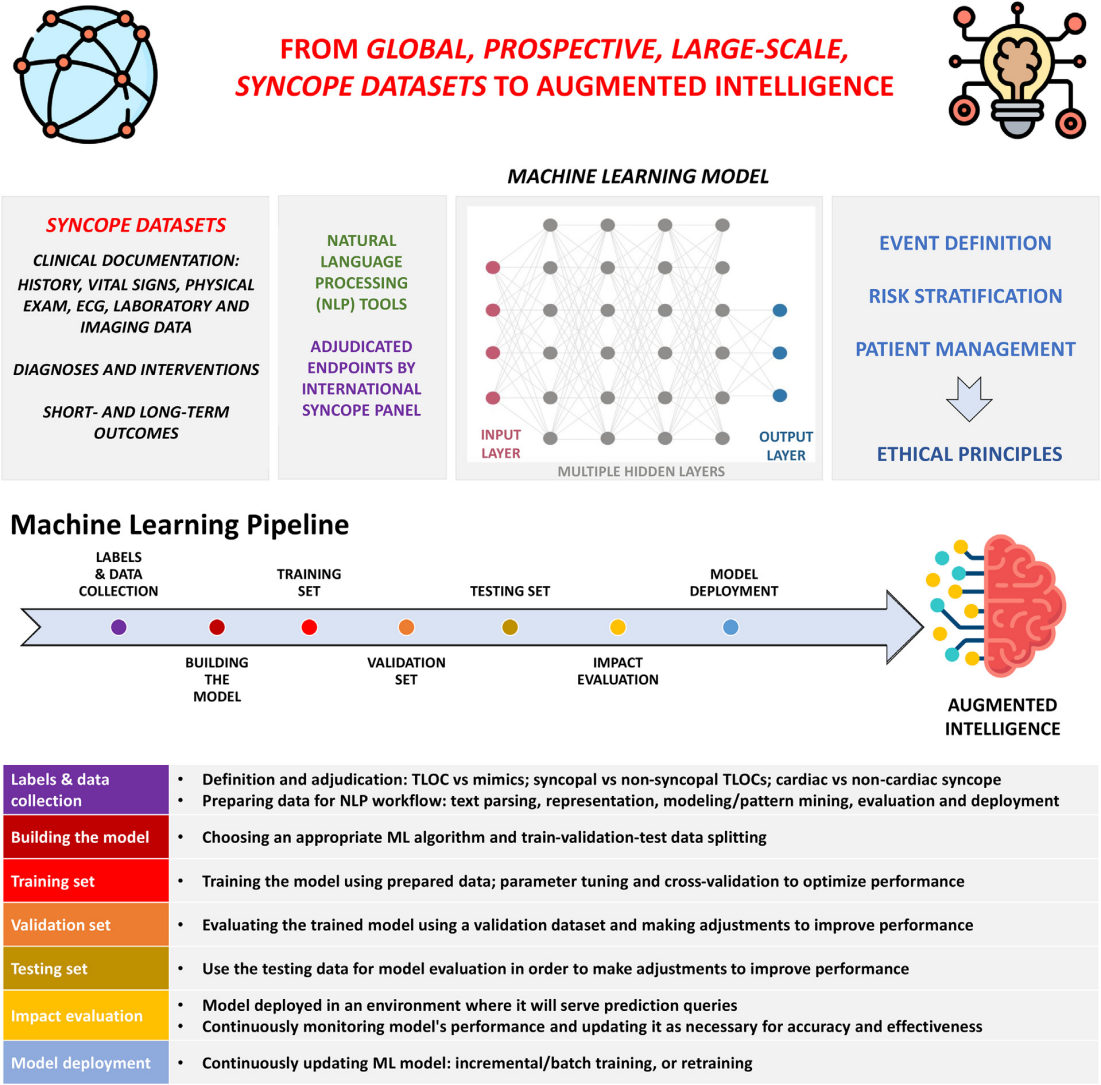
**Developing a ML Model for Syncope** Proposed steps to build a ML model for syncope. **(First row)** Emphasizing importance of large scale, prospective, and international datasets. **(Second row)** Potential inputs **(left)** and outputs **(right)** of a ML model to address the main clinical objectives in syncope management while recognizing underlying ethical principles. **(Third row)** ML pipeline showing sequential stages of a supervised ML project. **(Fourth row)** Descriptions of each stage in the ML pipeline. Created with Flaticon.com and Biorender.com. TLOC = transient loss of consciousness.

Figure 4 shows proposed steps to using AI to improve various facets of syncope management, including the ideal data collection strategy, potential inputs and outputs, as well as sequential stages of ML. It is important to realize the iterative nature of this process in which several models are serially tested and optimized. This allows for parameter tuning at each iteration to increase accuracy of the validation set (while avoiding overfitting), with eventual selection of the best model for implementation into an augmented intelligence platform. These models must be validated prior to widespread use, and because ML algorithms may not consider a patient’s preference regarding treatment or workup, they must be personalized and balanced with shared decision-making.

Pairing human physicians with powerful AI technology to make clinical decisions may create several medicolegal and ethical challenges. AI performs without an emotional basis which limits its intelligence. Moreover, ethics are not built into AI models yet. Presently, AI could serve as ‘an aid’ to clinical management but it would be hard to imagine that ML-based prediction algorithms and platforms (ie, IBM Watson) could ever supplant human judgment, especially when it comes to making complex clinical decisions. Collaboration between AI experts and human doctors could foster an optimal approach to patient care.

Physicians bear ultimate responsibility and liability for clinical decisions and management. Using AI in management decisions may lead to unintended consequences. AI may threaten patient preference, autonomy, safety, privacy, and confidentiality, and it is important for any AI-derived decision-making to be fair and free from discrimination. The American Medical Association recognizes that “addressing the added risk to patient privacy and confidentiality, parsing out the boundaries between the physician’s and machine’s role in patient care, and adjusting the education of future physicians to proactively confront the imminent changes in the practice of medicine” are important steps.^55^ Proper education of physicians and awareness of AI-related clinical risks may enhance compliance with regulations and assuage legal risks for health-care professionals and institutions.^56^

## Conclusions

AI shows potential in providing novel strategies to improve the care of syncope patients, but exactly how and to what degree, is presently unknown. This review article, based on a multidisciplinary international contingent, highlights the clinical objectives, current challenges, and potential solutions to using AI for the evaluation and management of syncope. No matter the clinical endpoints pursued, understanding the capabilities and ingredients for ML is essential. Patient-centered responsibility and liability for clinical decisions based on AI is paramount. The development of sizeable, high-quality datasets and clinically relevant ML models will require collaborative partnerships among clinicians, data scientists, medicolegal experts, and leaders in the field. Such collaboration should foster a reality where AI will *complement* rather than *compete* with the current state-of-the-art in syncope management.

## Funding support and author disclosures

This research was funded by the Iowa Initiative for Artificial Intelligence (IIAI), Carver College of Medicine Office of Research, University of Iowa. Dr Olshansky is on the Data and Safety Monitoring Board of AstraZeneca. Dr Sonka is an inventor and has patents and patent applications in computer vision and medical image analysis; and is a cofounder of Medical Imaging Applications, LLC, Coralville, Iowa, USA and VIDA Diagnostics, Inc, Coralville, Iowa, USA. Dr Venkatesh Thiruganasambandamoorthy is supported through a Physicians’ Services Incorporated Foundation Mid-Career Clinical Researcher award and University of Ottawa Tier-1 Clinical Research Chair in Cardiovascular Emergencies award. Dr Thiruganasambandamoorthy has received peer-reviewed grant funds for studies from the following governmental or non-profit agencies: the 10.13039/501100000241Physicians’ Services Incorporated Foundation—Ontario, Canada, 10.13039/501100000024Canadian Institutes of Health Research, 10.13039/100004411Heart and Stroke Foundation Canada, and the Cardiac Arrhythmia Network of Canada as part of the Networks of Centres of Excellence (NCE; and is a consultant for the NIH funded Practical Approaches to Care in Emergency Syncope (PACES) study. All other authors have reported that they have no relationships relevant to the contents of this paper to disclose.

## Supplementary data

Supplemental Appendices 1 and 2
