# Supplementary material for: Can Artificial Intelligence Enhance Syncope Management? A JACC: Advances Multidisciplinary Collaborative Statement
Source: JACC Adv. 2023 May 10;2(3):100323. doi: 10.1016/j.jacadv.2023.100323 (PMC11198330; doi:10.1016/j.jacadv.2023.100323)
Supplement: Supplemental Appendices 1 and 2 [file mmc1.pdf]

SUPPLEMENTAL APPENDIX

A Review of Select Syncope Risk Stratification Tools

Numerous syncope risk stratification tools have been developed for the ED setting.[1-13] The San Francisco Syncope Rule (SFSR) was one of the first decision rules developed to risk stratify patients and guide admissions.[7,14] It was a single site and prospectively derived study but had difficulty with external validation due to its unique definition of syncope and application of ECG criteria.[1,15-18]

The ROSE rule (Risk stratification Of Syncope in the Emergency department) was derived in 550 adult patients presenting to a United Kingdom ED with syncope, of whom 7.6% had a serious outcome or died within one month.[8] Again, it was validated at the same institution. The rule recommends admission to the hospital if the patient has any of the following risk factors: B-type natriuretic peptide (BNP) ≥300 pg/ml (odds ratio [OR] 7.3), positive fecal occult blood (OR 13.2), hemoglobin ≤90 g/l (OR 6.7), oxygen saturation ≤94% (OR 3.0), or Q-wave on the ECG (OR 2.8). Because the ROSE score requires measurement of BNP levels, it has only been subject to small external validation studies[19-21] and is currently only suitable for use in hospital or ED settings.

The Canadian Syncope Risk Score (CSRS) is one of the recently developed risk scores. The CSRS was derived and validated by enrolling 8176 patients from 11 Canadian EDs in whom no serious condition was identified during the index evaluation.[22,23] The tool predicted 30-day probability of various serious outcomes. CSRS scores range from -3 to +11 and allow patients to

be classified into very-low, low, medium, high, and very-high-risk strata with the observed risk of 30-day serious outcomes ranging from 0.3% to 42.7%.[24]

The tool provided the probability of specific outcome types (www.teamvenk.com/csrs), including death, ventricular arrhythmia, non-ventricular arrhythmia, and non-arrhythmic serious conditions, to aid ED management decisions. The study enrolled all adult patients with syncope for improved generalizability, excluded patients with serious conditions identified during the index ED evaluation, and included syncope-specific outcomes. There have been concerns about the need for a troponin for tool application,[25] and as a result, investigators have developed troponin criteria and included the subjective physician diagnostic impression predictor (vasovagal or cardiac syncope) which has been shown to be reliable and powerful.[26]

Currently, no syncope rule is widely used in EDs due to their poor predictive value or lack of external validation. Additionally, the multitude of objectives highlighted by these rules are varied and heterogeneous. The varying endpoints and shifting timelines call for improved clarity in the management of syncope, perhaps AI-enhanced techniques can help.

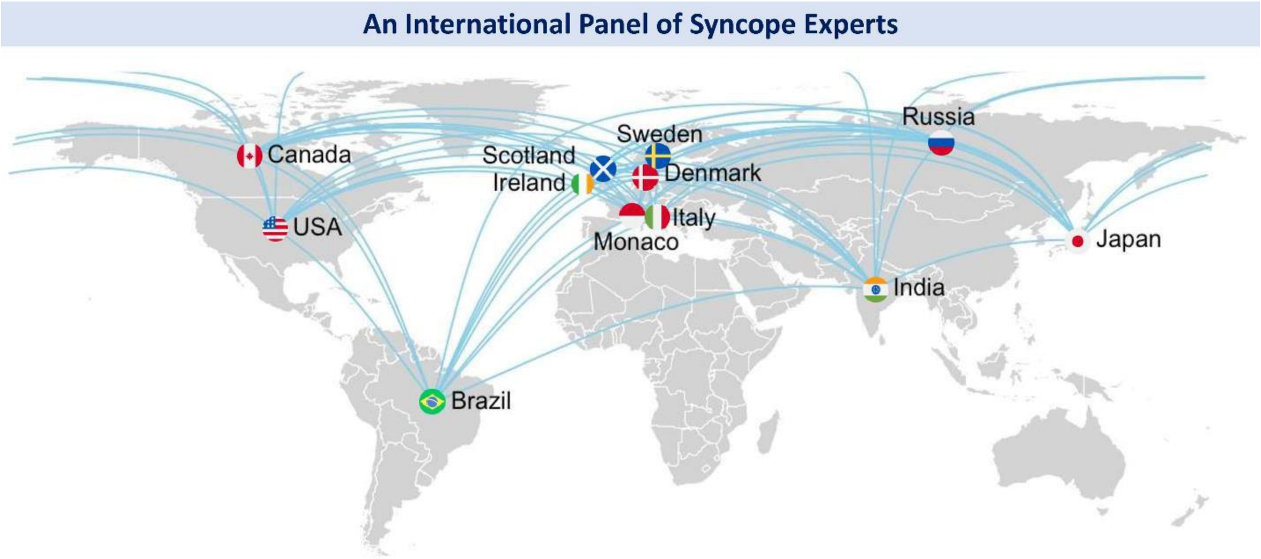

Supplemental Figure 1. An International Panel of Syncope Experts: World Map

A world map highlighting countries represented at the virtual meeting on September 25, 2021, which aimed to discuss the application of artificial intelligence in the field of syncope. The international panel included a total of 39 clinicians and investigators: including 25 syncope experts from 12 countries around the globe.

Supplemental Table 1: An International Panel of Syncope Experts

**North America**	
David Benditt, MD, FACC, FRCPC, FHRS	Cardiac Arrhythmia Center, Cardiovascular Division, University of Minnesota, Minneapolis, MN, USA
Rakesh Gopinathannair, MD, MA	Kansas City Heart Rhythm Institute, Overland Park, KS
Padma Kaul, PhD	Canadian Virtual Coordinating Center for Global Collaborative Cardiovascular Research Centre, University of Alberta, Edmonton, Alberta, Canada
Jeanne Poole, MD	Division of Cardiology, University of Washington, Seattle, WA, USA
James Quinn, MD, MS	Division of Emergency Medicine, Stanford University, Stanford, CA, USA
Roopinder Sandhu MD, MPH	University of Alberta, Edmonton, Alberta, Canada
Robert Sheldon, MD, PhD	Department of Cardiac Sciences, University of Calgary, Calgary, Alberta, Canada
Win-Kuang Shen, MD	Department of Cardiovascular Diseases, Mayo Clinic Arizona Phoenix, AZ, USA

Benjamin Sun, MD, MPP	Department of Emergency Medicine, Oregon Health and Science University, Portland, OR, USA
Venkatesh Thiruganasambandamoorthy, MBBS, CCFP-EM, MSc	Department of Emergency Medicine, University of Ottawa, Ottawa, Ontario, Canada
**Europe**	
Michele Brignole, MD, FESC	Department of Cardiology, S. Luca Hospital, IRCCS, Istituto Auxologico Italiano, Milan, Italy
Giorgio Costantino, MD	Fondazione IRCCS Ca' Granda, Ospedale Maggiore Policlinico<br>Università degli Studi di Milano<br>Milan, Italy
Franca Dipaola, MD	Internal Medicine, Syncope Unit, IRCCS Humanitas Research Hospital, Rozzano; Humanitas University, Pieve Emanuele, Milan, Italy
Artur Fedorowski, MD, PhD	Department of Cardiology, Karolinska University Hospital, Stockholm, Sweden
Raffaello Furlan, MD	Internal Medicine, Syncope Unit, IRCCS Humanitas Research Hospital, Rozzano; Department of Biomedical Sciences,

	Humanitas University, Pieve Emanuele, Milan, Italy
Mauro Gatti, PhD	Active Intelligence Center, IBM, Bologna, Italy
Madeleine Johansson, MD, PhD	Department of Cardiology, Skåne University Hospital, Lund University, Malmo, Sweden
Rose Anne Kenny, MD, FRCP, FRCPI, FRCPEdin, FESC, FTCD, FFPHMI (Hon), MRIA,D.Sc.h.c.	Falls and Syncope Unit, Mercer's Institute for Successful Ageing, St James's Hospital, Dublin, Ireland
Alessandro Giaj Levra, MD	Department of Biomedical Sciences, Humanitas University, Milan, Italy
Roberto Menè, MD	1. Heart Rhythm Department, Clinique Pasteur, Toulouse, France 2. Department of Medicine and Surgery, University of Milano-Bicocca, Milan, Italy
Matthew J Reed MB, MD	1. Emergency Medicine Research Group Edinburgh (EMERGE), Department of Emergency Medicine, Royal Infirmary of Edinburgh, Edinburgh, UK. 2. Acute Care Group, Usher Institute of Population Health Sciences and Informatics, College of Medicine and Veterinary Medicine, University of Edinburgh, Edinburgh, UK

Fabrizio Ricci MD, PhD, MSc	Department of Neurosciences, Imaging and Clinical Sciences, Institute for Advanced
Martin Ruwald, MD, PhD	Department of Cardiology, Copenhagen University Hospital, Herlev and Gentofte Hospital, Hellerup, Denmark
Richard Sutton, MB BS DSc FACC	Department of Cardiology, Hammersmith Hospital Campus, Imperial College, London, UK
**Asia**	
Haruhiko Abe, MD, PhD	Department of Heart Rhythm Management, University of Occupational and Environmental Health, Kitakyushu, Japan
Dmitry Lebedev, MD, PhD, DSc	Arrhythmology Department, Almazov National Medical Research Center, St. Petersburg, Russia
Tamara Lyubimtseva, MD, PhD	Arrhythmology Department, Almazov National Medical Research Center, St. Petersburg, Russia
Hygriv Rao, MBBS, MD	Division of Pacing and Electrophysiology, KIMS Hospitals, Hyderabad, Telangana, India

Jayaprakash Shenthar MBBS, MD	Sri Jayadeva Institute of Cardiovascular Sciences and Research, Bangalore, Karnataka, India
**South America**	
Denise Hachul, MD, PhD	Heart Institute, University of Sao Paulo Medical School, Sao Paulo, Brazil

Names and affiliations of the clinicians and investigators from around the world who participated in a virtual meeting on September 25, 2021, that highlighted the potential application of artificial intelligence to the management of syncope and inspired this manuscript. Among them were 25 syncope experts from 12 countries across 4 continents. A small few were not able to attend the meeting but contributed written statements on the subject of artificial intelligence and syncope.

Supplemental Table 2. Select Viewpoints on AI[a] from an International Panel of Syncope Experts

Question	Expert Viewpoint
Should we be afraid of AI?	*Dr. Mauro Gatti, Italy:* We should not be afraid of AI. It has been around since the fifties and human beings are still here. The key point is to study "the human in the loop," namely, the interaction between the clinician and the AI tool. AI can be used for automation or augmentation; medicine is no exception. It may be reasonably expected that automation will ultimately be applied to works that are simple for humans, while augmentation will be applied to more complex activities. Several aspects of current AI models may make them more suitable for augmentation than automation, notably robustness, interpretability, and accountability.<br>The use of an AI model for augmentation has its own specific challenges. The two most critical requirements of a clinical decision support system to enable effective utilization are: 1. fitting into the clinical workflow, and 2. trustworthiness. Trust is known to be difficult to build and easy to destroy. Human-in-the-loop is a broad area of research that aims at better understanding the role of the human in the entire life cycle of an AI model's design, development, and utilization.
How can AI be helpful in syncope management?	*Alessandro Giaj Levra, Italy:* AI algorithms can recognize and classify syncope within huge amounts of administrative data with significant time

[a] AI, artificial intelligence

	reduction and remarkable accuracy[27] compared with manual revision and ICD[b]-9 and ICD-10 automatic methods.[28]<br><br>Use of these algorithms to autonomously identify and classify syncope patient characteristics from EMR[c]s would permit the comparison of a single patient's clinical features to others with the same risk factors and whose outcomes are known. Therefore, AI may help emergency physicians personalize the risk of patients with syncope.<br><br>In clinical practice, such an approach would provide valuable information for the ED[d] physician's decision making, such as hospital admission or discharge. Furthermore, the possibility to promptly identify high risk patients may enable preemptive interventions to avoid long-term complications or unnecessary exams, thus optimizing resource allocation. From an *outpatient* perspective, thanks to the increasing use of wearables, AI could provide real-time risk stratification during daily life by making an early analysis of vital parameters obtained before a syncope event.<br><br>Needless to say, application of AI in syncope diagnosis, prognosis, and management can become a paradigm to be used in the ED for other disorders such as chest pain, abdominal pain, dyspnea of unknown origin among others.

[b] ICD, international classification of diseases
[c] EMR, electronic medical record
[d] ED, emergency department

How can AI be used to improve syncope management in the ED?	*Dr. Roberto Mene, Italy:* AI may facilitate individualized patient-level risk prediction. Triaging is a key process for proper patient management and resource allocation at the time of ED admission and discharge. AI-based algorithms may automatically extract and analyze data contained in ED annotations and biologic recordings (e.g., vital parameters, [e]ECG monitoring). Although successful examples of this application already exist,[29] further validation on large patient cohorts is needed. AI may help recognize the etiology of syncope. AI-based diagnosis support systems may formulate diagnoses with an accuracy comparable to expert clinicians.[30] Regarding syncope, algorithms have been proposed to identify and differentiate it from other causes of TLOC,[f] [27,31] but AI-based methods to identify its etiologic mechanism are still missing.<br>From a practical standpoint, embedding AI-based prediction algorithms into ED patient management software may provide physicians with real-time assistance based on data entered into the patient EMR.
How can AI be used to improve overall syncope management?	*Dr. Roopinder Sandhu, Canada:* A major contribution of AI/ML[g] may lie in improving risk stratification of syncope patients at point of care, whether in the ambulance or in the ED. Diagnostic uncertainty and unclear prognoses confound syncope triage and risk assessment. Differences in management, healthcare costs, and outcomes among patients presenting with syncope to the ED who were discharged home

[e] ECG, electrocardiogram
[f] TLOC, transient loss of consciousness
[g] ML, machine learning

	versus admitted to the hospital have been established.[32] This latter group has been stratified into those with and without an established syncope etiology.<br>In Canada's universal health care system, opportunities for efficient and cost-effective care delivery are preeminent. Accordingly, AI/ML models have been considered based on longitudinal data from population-level administrative databases including inpatient, outpatient, ED, centralized laboratory results, pharmaceutical claims, and vital statistics to identify syncope patients who are at high risk for adverse events such as hospitalizations and mortality. More recently, we have linked digital ECG data to further enhance these AI models. An international collaboration will provide unique opportunities to validate algorithms in different health care settings but also to examine effectiveness in future prospective clinical trials.
What are the opportunities and future directions in the use of AI for improving syncope management?	*Dr. Denise Hachul, Brazil*: Considering syncope, we are faced with paroxysmal symptoms representing numerous medical disorders, in isolation or association, which confounds diagnostic acumen. Establishing whether TLOC is syncope, stratifying its risk and defining the etiology to assure the best treatment remains a great challenge. Having time for long consultations with a team of dedicated multidisciplinary experts may be supplanted by AI/ML. However, patients with syncope need to be heard and physically examined, which can be time-consuming. Our experience in a dedicated syncope unit over

	the last 30 years, with a team of committed professionals, has shown that the more you practice, the more time you spend, and the more diverse the team is, the better the outcome for the patient. Further, public medical services, especially in less developed countries, have very poor conditions of care and this model is difficult to reproduce.[33] AI is a promising tool to accelerate the process of algorithmic application to normalize clinical care worldwide, reducing diagnostic errors and improving patient outcomes.<br>At a time when the quality of doctor-patient relationships are assessed, the lack of physical examination is criticized, and a more humanized approach is requested. On the contrary, the importance of the computer in medicine and public health is increasing. Not only because of the accelerated growth of medical knowledge, but especially the development of technologies to support clinical decisions, which should be used cautiously to provide more diagnostic accuracy.[34]<br>Integrative medicine has benefited greatly from data storage, which has increased exponentially in recent years, creating the concept of big data. Supercomputers can create true neural networks and data processing algorithms in several areas, providing increasingly accurate diagnostic hypotheses.[34,35] This data can be reviewed by specialists all over the world, with unprecedented advantages for health services and patient care.

	TLOC and syncope are among the symptoms that would most benefit from this new tool, considering the vast possibilities of diagnosis and the need of faster and more accurate solutions.[36]<br><br>AI will make it possible to spread the knowledge we have learned over the years and enable the development of new routes for better patient care.
How can AI/ML make the right clinical decision?	*Dr. Fabrizio Ricci, Italy*: Leveraging unsupervised ML-based cluster analysis and phenomapping techniques[37,38] might help recognize covert disease heterogeneity in patients with unexplained syncope and identify subsets - phenogroups - with distinct pathophysiological profiles and differential outcomes. Clustering is the most common unsupervised learning technique for exploratory data analysis to find hidden patterns or groupings in data according to similarity.[39]<br><br>NLP[h] is a high-throughput technology that can be leveraged to automatically extract, classify, and label elements from massive volumes of raw, unstructured, and unlabeled datasets. Large-scale NLP-driven analysis of unstructured EMR in combination with AI-powered deep phenotyping techniques would be a desirable approach to identify unique patterns of association among phenotypic variables at scale and discover subgroups of patients with clinically distinct phenotypic and prognostic profiles. Ultimately, whether the identification of distinct phenogroups

[h] NLP, natural language processing

	can impact clinical decision making or deliver true patient benefit will have to be critically appraised.[38,40]
What are the future directions of personalized medicine and AI ?	*Dr. Franca Dipaola, Italy*: In the age of precision medicine, clinicians' decision-making is asked to rely upon personalized risk predictions. For syncope, risk stratification tools based on traditional statistical methods failed to be superior to simple clinical judgment,[41,42] thus requiring the use of alternative methodologies[36] and innovative technologies. [27]<br>The possibility to automatically classify and analyze risk factors from large patient populations by ML algorithms could generate significant progress in individual risk stratification. In particular, since most of the prognostic information is available in textual form, the use of NLP techniques could allow the automatic extraction of relevant phenotypes from EMR or ad hoc repositories.<br>Implementation of these techniques, through data sharing, can promote the optimization of syncope patients' diagnostic work up and personalization of care.[27]
What are the most important first steps in developing an AI-based syncope project?	*Dr. Benditt, USA:* The use of AI is so critically dependent on the training set. And the training set has to be as good as it can possibly be. The difficulty we have is we don't have a training set where all 20 of us would all agree on the diagnosis. These datasets have all the limitations of the most component of us as syncope people. Consequently, AI can never be better than that because the training set is limited by our own ability to know the diagnosis. We can use it for individual things like

	identifying the vasovagal faint. But in the complex world of syncope, with many different diagnoses and with no gold standard every one of us would agree on, we're going to have difficulties. We need that gold standard. How to get that is a major challenge. We have to be realistic in our limitations in AI development without a solid gold standard.<br>We need to develop detailed smart questionnaires and then test them to ascertain the predictive accuracy for various diagnoses. This is a complex task. Perhaps weaning out the most common diagnostic categories would be a feasible first step. Accomplishing this will necessitate time and substantial funding. Perhaps an NIH[i] grant should be embarked upon.<br>*Dr. Tamara Lyubimtseva, Russia*: To date, for this team, it is necessary to collect big clinical material by way of a special cloud with mathematical data processing.
How should we approach data acquisition?	*Dr. Robert Sheldon, Canada*:<br>#1: Data acquisition and analysis efforts should focus on cause of syncope as well as risks incurred from syncope and independent comorbidities.<br>#2: The first tier of effort should target common and problematic syncope mimics: convulsive syncope versus epileptic convulsions, falls versus faints in the older patient, or collapse without apparent biological cause.

[i] NIH, National Institute of Health

	#3: The second tier of effort should target risk stratification. This should start with agreement on whether the primary outcome should be related to the cause of syncope, or simply be a composite of poor outcomes due to the range of comorbidity factors and frailty. This effort should include sufficient power to be statistically robust.<br>#4: Scrupulous care must be taken to collect patients with gold standard diagnoses to initiate a valid AI assessment. This will require a large prospectively collected population and not administrative data.<br>#6: AI/ML modelling should be done in sequential tiers or using a prior stratified patient populations of adequate size.
How can we apply AI to the ECG in syncope evaluation?	*Dr. Artur Fedorowski, Sweden:* How AI-ECG interpretation is used depends on the clinical setting—in a busy ED where time is short and risks should be minimized, in primary care where physicians deal with an array of medical conditions and may lack an experienced eye, or in cardiology where a syncope specialist may need a fine-tuned ECG assessment to detect an underlying channelopathy or conduction disorder. Each clinical context and its available resources would dictate the use of this technology.
What are some unique applications for AI in syncope?	*Additional solutions from the University of Iowa multidisciplinary team:*<br>*Dr. Milena Gebska, USA:* One potential solution may be electronic "syncope check-in booths," involving a custom set of questions created based on supervised learning experiences that the patient, family member, or witness would be asked to complete in the ED. This could

	potentially help uncover "the truth," and streamline the initial triage, evaluation, and assessment of these patients while simultaneously contributing to an augmented intelligence platform.<br>*Avinash Mudireddy, USA:* AI-derived data collection processes could be created via speech, vision, or text. They could be refined over time through re-training on new data, leading to highly developed syncope-specific AI.

This table contains select written and quoted viewpoints from the international panel of syncope experts in regard to specific objectives and opportunities for AI in syncope research.

Supplemental References

1. Birnbaum A, Esses D, Bijur P, Wollowitz A, Gallagher EJ. Failure to validate the San Francisco Syncope Rule in an independent emergency department population. Ann Emerg Med 2008;52:151-9.
2. Costantino G, Perego F, Dipaola F et al. Short- and long-term prognosis of syncope, risk factors, and role of hospital admission: results from the STePS (Short-Term Prognosis of Syncope) study. J Am Coll Cardiol 2008;51:276-83.
3. Daccarett M, Jetter TL, Wasmund SL, Brignole M, Hamdan MH. Syncope in the emergency department: comparison of standardized admission criteria with clinical practice. Europace 2011;13:1632-8.
4. Del Rosso A, Ungar A, Maggi R et al. Clinical predictors of cardiac syncope at initial evaluation in patients referred urgently to a general hospital: the EGSYS score. Heart 2008;94:1620-6.
5. Martin GJ, Adams SL, Martin HG, Mathews J, Zull D, Scanlon PJ. Prospective evaluation of syncope. Ann Emerg Med 1984;13:499-504.
6. Mechanic OJ, Pascheles CY, Lopez GJ et al. Using the Boston Syncope Observation Management Pathway to Reduce Hospital Admission and Adverse Outcomes. West J Emerg Med 2019;20:250-255.
7. Quinn JV, Stiell IG, McDermott DA, Sellers KL, Kohn MA, Wells GA. Derivation of the San Francisco Syncope Rule to predict patients with short-term serious outcomes. Ann Emerg Med 2004;43:224-32.

8. Reed MJ, Newby DE, Coull AJ, Prescott RJ, Jacques KG, Gray AJ. The ROSE (risk stratification of syncope in the emergency department) study. J Am Coll Cardiol 2010;55:713-21.

9. Schladenhaufen R, Feilinger S, Pollack M, Benenson R, Kusmiesz AL. Application of San Francisco Syncope Rule in elderly ED patients. Am J Emerg Med 2008;26:773-8.

10. Serrano LA, Hess EP, Bellolio MF et al. Accuracy and quality of clinical decision rules for syncope in the emergency department: a systematic review and meta-analysis. Ann Emerg Med 2010;56:362-373 e1.

11. Thiruganasambandamoorthy V, Hess EP, Turko E, Perry JJ, Wells GA, Stiell IG. Outcomes in Canadian emergency department syncope patients--are we doing a good job? J Emerg Med 2013;44:321-8.

12. Thiruganasambandamoorthy V, Stiell IG, Sivilotti ML et al. Risk stratification of adult emergency department syncope patients to predict short-term serious outcomes after discharge (RiSEDS) study. BMC Emerg Med 2014;14:8.

13. Thiruganasambandamoorthy V, Taljaard M, Stiell IG et al. Emergency department management of syncope: need for standardization and improved risk stratification. Intern Emerg Med 2015;10:619-27.

14. Quinn J, McDermott D, Stiell I, Kohn M, Wells G. Prospective validation of the San Francisco Syncope Rule to predict patients with serious outcomes. Ann Emerg Med 2006;47:448-54.

15. Quinn J, McDermott D. ECG criteria of the San Francisco Syncope Rule. Ann Emerg Med 2011;57:72-3; author reply 73.

16. Quinn J, McDermott D. Electrocardiogram findings in emergency department patients with syncope. Acad Emerg Med 2011;18:714-8.

17. Thiruganasambandamoorthy V, Hess EP, Alreesi A, Perry JJ, Wells GA, Stiell IG. External validation of the San Francisco Syncope Rule in the Canadian setting. Ann Emerg Med 2010;55:464-72.

18. McDermott D, Quinn J. Response to "failure to validate the San Francisco Syncope Rule in an independent emergency department population". Ann Emerg Med 2009;53:693; author reply 693-4.

19. Patel A, Fakorede F, Waldman B, Elbaum P, Patel A, Iliadis E. Abstract 120: ROSE Rule is Underutilized in the Emergency Department Evaluation of Syncope. Circulation; https://doiorg/101161/circoutcomes5suppl_1A120 2018.

20. Thiruganasambandamoorthy V, Ramaekers R, Rahman MO et al. Prognostic value of cardiac biomarkers in the risk stratification of syncope: a systematic review. Intern Emerg Med 2015;10:1003-14.

21. Reed MJ, Henderson SS, Newby DE, Gray AJ. One-year prognosis after syncope and the failure of the ROSE decision instrument to predict one-year adverse events. Ann Emerg Med 2011;58:250-6.

22. Thiruganasambandamoorthy V, Kwong K, Wells GA et al. Development of the Canadian Syncope Risk Score to predict serious adverse events after emergency department assessment of syncope. CMAJ 2016;188:E289-E298.

23. Thiruganasambandamoorthy V, Sivilotti MLA, Le Sage N et al. Multicenter Emergency Department Validation of the Canadian Syncope Risk Score. JAMA Intern Med 2020;180:737-744.

24. Thiruganasambandamoorthy V, Yan JW, Rowe BH et al. Personalised risk prediction following emergency department assessment for syncope. Emerg Med J 2021.

25. Egerton-Warburton D, Maclean P, Cullen L. Letter by Egerton-Warburton et al Regarding Article, "Duration of Electrocardiographic Monitoring of Emergency Department Patients With Syncope". Circulation 2019;140:e654.

26. Toarta C, Mukarram M, Arcot K et al. Syncope Prognosis Based on Emergency Department Diagnosis: A Prospective Cohort Study. Acad Emerg Med 2018;25:388-396.

27. Dipaola F, Gatti M, Pacetti V et al. Artificial Intelligence Algorithms and Natural Language Processing for the Recognition of Syncope Patients on Emergency Department Medical Records. J Clin Med 2019;8.

28. Furlan L, Solbiati M, Pacetti V et al. Diagnostic accuracy of ICD-9 code 780.2 for the identification of patients with syncope in the emergency department. Clin Auton Res 2018;28:577-582.

29. Costantino G, Falavigna G, Solbiati M et al. Neural networks as a tool to predict syncope risk in the Emergency Department. Europace 2017;19:1891-1895.

30. Baker A, Perov Y, Middleton K et al. A Comparison of Artificial Intelligence and Human Doctors for the Purpose of Triage and Diagnosis. Front Artif Intell 2020;3:543405.

31. Wardrope A, Jamnadas-Khoda J, Broadhurst M et al. Machine learning as a diagnostic decision aid for patients with transient loss of consciousness. Neurol Clin Pract 2020;10:96-105.

32. Sandhu RK, Tran DT, Sheldon RS, Kaul P. A Population-Based Cohort Study Evaluating Outcomes and Costs for Syncope Presentations to the Emergency Department. JACC Clin Electrophysiol 2018;4:265-273.

33. Brignole M, Moya A, de Lange FJ et al. 2018 ESC Guidelines for the diagnosis and management of syncope. Eur Heart J 2018;39:1883-1948.

34. Sun JY, Shen H, Qu Q, Sun W, Kong XQ. The application of deep learning in electrocardiogram: Where we came from and where we should go? Int J Cardiol 2021;337:71-78.

35. Miller DD, Brown EW. Artificial Intelligence in Medical Practice: The Question to the Answer? Am J Med 2018;131:129-133.

36. Dipaola F, Shiffer D, Gatti M, Mene R, Solbiati M, Furlan R. Machine Learning and Syncope Management in the ED: The Future Is Coming. Medicina (Kaunas) 2021;57.

37. Segar MW, Patel KV, Ayers C et al. Phenomapping of patients with heart failure with preserved ejection fraction using machine learning-based unsupervised cluster analysis. Eur J Heart Fail 2020;22:148-158.

38. Shah SJ, Katz DH, Selvaraj S et al. Phenomapping for novel classification of heart failure with preserved ejection fraction. Circulation 2015;131:269-79.

39. Dey D, Slomka PJ, Leeson P et al. Artificial Intelligence in Cardiovascular Imaging: JACC State-of-the-Art Review. J Am Coll Cardiol 2019;73:1317-1335.

40. Vollmer S, Mateen BA, Bohner G et al. Machine learning and artificial intelligence research for patient benefit: 20 critical questions on transparency, replicability, ethics, and effectiveness. BMJ 2020;368:l6927.

41. Dipaola F, Costantino G, Perego F et al. San Francisco Syncope Rule, Osservatorio Epidemiologico sulla Sincope nel Lazio risk score, and clinical judgment in the assessment of short-term outcome of syncope. Am J Emerg Med 2010;28:432-9.

42. Solbiati M, Quinn JV, Dipaola F et al. Personalized risk stratification through attribute matching for clinical decision making in clinical conditions with aspecific symptoms: The example of syncope. PLoS One 2020;15:e0228725.
